# Barriers and facilitators at pre-implementation of a type 2 diabetes screening program in dental care using the consolidated framework for implementation research and the theoretical domains framework

**DOI:** 10.1186/s12913-026-14902-7

**Published:** 2026-06-06

**Authors:** Vanessa Y. Hiratsuka, Diane King, Gretchen M. Day, Kathryn Koller, Todd Takeno, Barbara Stillwater, Julie A. Beans

**Affiliations:** 1 Southcentral Foundation, Research Department, Anchorage, 99508 AK USA; 2 Center for Behavioral Health Research and Services, University of Alaska Anchorage, Anchorage, 99508 AK USA; 3 Alaska Native Tribal Health Consortium, Research Services, Anchorage, 99508 AK USA; 4 Anchorage, AK USA

**Keywords:** Oral health, Diabetes, American Indian, Alaska Native, Barriers, Facilitators, CFIR

## Abstract

**Background:**

Screening and referral for type 2 diabetes mellitus (T2DM) during dental care visits has the potential for expanding preventive care. Using the consolidated framework for implementation research (CFIR) and the theoretical domains framework (TDF), we examined the barriers and facilitators at pre-implementation of a community-driven T2DM screening program in an urban dental clinic serving Alaska Native and American Indian (AN/AI) adults.

**Methods:**

This convergent mixed-methods parallel study was informed by the updated CFIR. Data were collected in 2023 through a 13-item survey of adult AN/AI potential recipients of the T2DM screening innovation/intervention, and individual in-person interviews with dental and primary care providers, staff, and operational leaders who were from the population of potential innovation deliverers. Univariate statistics and differences between strata were analyzed using R software. Interview transcripts were coded onto CFIR and TDF domains using template analysis then thematically analyzed. A convergent analysis identified areas of convergence, divergence, or complementarity.

**Results:**

Two hundred and fifty potential innovation recipients provided survey responses. The majority of survey respondents agreed that the dental clinic is a good place to get T2DM screening, thought screening would be helpful, and had no concerns about the setting. Some respondents had concerns about T2DM screening in the dental setting or by dental staff due to T2DM screening not usually occurring in a dental visit. However, most survey respondents thought the dental clinic as a good place to get screened for diabetes and had low levels of concern about T2DM screening in dental settings. Primary care providers did not see the need for T2DM screening in dental settings; however, about half of potential innovation recipients thought the T2DM screening information would be helpful for their doctor and would be a good way to find if they were at risk for or currently had T2DM.

**Conclusions:**

Using CFIR and TDF, we identified barriers and facilitators to inform the design of a pilot process, development of pilot materials, and selection of innovation deliverers.

**Supplementary Information:**

The online version contains supplementary material available at 10.1186/s12913-026-14902-7.

**Table:** 

Contributions to the literature	
• This formative research is the first to involve medical and dental health care providers and community members in a Tribal health setting using theoretical frameworks to identify barriers and facilitators at pre-implementation to develop a future dental based intervention.	
• This study illustrates the application of implementation science in an Indigenous health system to address a community health priority and implementation gap.	
• Identification of relevant barriers and facilitators using both the theoretical domains framework and the updated consolidated framework for implementation research was supportive in design of a pilot process, development of pilot materials and selection of innovation deliverers.	

## Introduction

Effective delay of onset and prevention of type 2 diabetes mellitus (T2DM) requires offering T2DM screening in healthcare settings that routinely serve populations with known risk factors [1]. Expanding T2DM screening in ways that increase access may improve the reach of T2DM preventive services for individuals unaware of their current risk-level.

T2DM is the fifth leading cause of Alaska Native and American Indian (AN/AI) mortality for both men and women, in all adult age groups [2]. Findings from the Alaska Education and Research Towards Health (EARTH) baseline cohort study documented the prevalence of modifiable risk factors contributing to T2DM progression (i.e., tobacco use [3], obesity [3, 4], large waist circumference [4], gestational diabetes [5], and physical inactivity [6]) among AN/AI study participants between 2004 and 2006. A follow-up study conducted with an urban subset of EARTH participants during 2015–2017 [7] calculated T2DM incidence of 16.5/1,000 person-years (PY) and pre-diabetes incidence at 77.6/1,000 PY [8]. T2DM prevalence (12% at baseline in 2004–2006) had increased to 25% and pre-diabetes prevalence (31% at baseline) had risen to 70% in just 10 years [8]. Despite improved T2DM screening since baseline measures were obtained, pre-diabetes diagnosis, referral, and treatment are needed to effectively delay or prevent T2DM onset and sequalae.

Successful intervention development requires pre-implementation engagement with stakeholders, including input from beneficiaries (care recipients) and deliverers (care providers) of a proposed innovation to proactively co-develop a sustainable and effective design process [9, 10]. Central to development and tailoring of an intervention is the alignment of potential innovation recipients and deliverers preferences [11, 12]. Due to the novelty of screening for T2DM in an existing AN/AI serving dental clinic, we sought to understand the influences on dental and primary care staff, operational leaders, and patients in the context in which they occur. We used the Consolidated Framework for Implementation Research (CFIR) and Theoretical Domains Framework (TDF) to provide theoretical guidance in the examination of barriers and facilitators to the innovation [13–15]. This manuscript describes the feedback from potential innovation recipients (survey respondents) and deliverers (interviewees) to be used to inform the design of a dental clinic placed T2DM screening innovation in an urban Tribal health organization.

## Methods

We used a convergent mixed methods parallel design [16] to understand receptivity to screening and referral for T2DM during a dental visit, and potential facilitators and barriers to implementing the T2DM screening and referral service as part of an oral health visit. If testing had not been performed within the past year, proposed screening included collecting individual patient information on T2DM risk factors and offering an HbA1c test by finger prick using a portable point-of-care analyzer within the dental clinic setting.

### Theoretical framework

The design of the project [17] was informed by community-based participatory research [18] and more specifically, Tribally-driven participatory research [19] approaches to acknowledge and honor potential innovation recipient and deliverer views and values in pre-implementation efforts. Furthermore, this study is grounded in key principles from Indigenous Standpoint Theory that emphasizes prioritizing Indigenous knowledge and agency, including leadership from AN/AI investigators, a focus on health issues (i.e., access to healthcare, oral health, T2DM) of high priority to AN/AI peoples, co-design with community partners, and potential for direct benefit to the community [20]. The study supports the community defined health priority of diabetes prevention and coordination of care across the healthcare system [21], builds on the strengths and resources within the community, involves community members in all phases of the research project (i.e., oversight through the research review process [22], collaborative partnership through the Planning Team, and maintenance of a long-term commitment by the research team). The overall study design builds on prior research exploring the potential to conduct T2DM screening and referral during dental appointments, particularly for patients who do not regularly access primary care [23–28], and adapts evidence-informed recommendations for the Tribal health setting.

The study design and data collection instruments were guided by the updated Consolidated Framework for Implementation Research (CFIR) [15, 29] to identify the facilitators and barriers at the organizational level: innovation characteristics (i.e., the thing being implemented), individual characteristics (i.e., roles and characteristics of those directly or indirectly delivering or receiving the innovation), inner setting (i.e., the environment in which the innovation is implemented), outer setting (i.e., the environment in which the inner setting exists), and implementation process (i.e., activities and strategies used to enact the innovation) domains (Table 1). The Theoretical Domains Frameworks (TDF) is an evidence-based tool consisting of 14 domains covering the main facilitators and barriers at the individual level: knowledge; intentions; skills; social/professional role and identity; beliefs about capabilities; beliefs about consequences; goals; reinforcement; memory, attention, and decision processes; optimism; environmental context and resources; social influences; emotion; and behavioral regulation [13, 30].

**Table 1:** Theoretical domains framework (TDF) and consolidated framework for implementation research (CFIR) domains and constructs used in surveys and interviews

TDF domain/ CFIR antecedent assessment	CFIR domains/ constructs	C-O survey item(s)	Provider interview question(s)
Knowledge; skills	Individuals-capability	• Would you like to receive a handout explaining diabetes and signs/risk factors?	• Is the relationship between oral health and diabetes recognized by dental professionals?
Social/ professional role and identity	Individuals- need	• It is important to get screened for diabetes.	• Screening encompasses several steps, including asking the C-O about their diabetes status using a questionnaire, reviewing the questionnaire to determine whether to do a diabetes screening blood test, collecting and analyzing the blood sample, and then providing a referral and documenting the results in the patient record. Could or should different people be involved in these steps? • Given that screening results may identify those currently at risk for diabetes as well as those with probable diabetes, who in your practice would be best positioned to relay the results, provide education, and coordinate next steps?
Beliefs about capabilities; optimism	Individuals- motivation; appropriateness	• How would you rate the helpfulness in being screened for diabetes in the dental setting? • Do you have concerns about getting diabetes testing during a dental visit?	not in guide
Beliefs about consequences	Individuals- need	• The following are common benefits for getting diabetes screening at the dental clinic. Which of the following benefits are most important to you?	not in guide
Reinforcement	Inner setting-incentive systems	not in survey	not in guide
Intentions; goals	Inner setting- culture	• How would you rate your level of concern in being screened for diabetes in the dental setting?	• Does the SCF Dental currently screen for chronic disease? • What competing priorities do you see for implementing diabetes screening and referrals in the dental clinic?
Memory, attention and decision processes; Social influences	Inner setting- relative priority; implementation climate	• not in survey	• Can you describe the protocol in place for referral to primary care? • What quality indicators do you currently use to monitor and make improvements to service delivery as well as C-O outcomes (e.g., chart review, dashboard reports, staff, and C-O feedback)?
Environmental context and resources	Inner setting- recipient-centeredness; implementation climate	The dental clinic is a good place to get screened for diabetes.	• Does SCF Dental support individuals with diabetes currently? • Whose buy-in will be necessary to successfully implement diabetes screening and referral in SCF dental clinics? • What changes might be necessary to implement prior to introducing a new screening protocol (e.g., documentation, billing, policies and procedures, reporting)? • Describe what you see as the main challenges to implementing diabetes screening as a part of routine dental care?
Emotion	Acceptability; implementation readiness	not in survey	• Are there any other considerations that should be kept in mind or addressed prior to, or as part of, the diabetes screening pilot project?
Behavioral regulation	Feasibility; implementation readiness	not in survey	• Would adding diabetes screening and referral to primary care be feasible for SCF dental clinic to do now? Why or why not?

### Setting

Southcentral Foundation (SCF), a Tribal healthcare organization, transitioned to a primary care patient centered medical home model in 1999. SCF utilizes integrated care teams to provide comprehensive health and wellness services to more than 70,000 AN/AI people living in south central Alaska [31]. SCF refers to patients as “customer-owners”. SCF Dental transitioned to a dental home model in 2023 [32], and utilizes dental care teams to provide oral health services to the SCF service population. The SCF dental and primary care closed system provides a uniquely viable setting to expand T2DM screening and referral for periodontal and other dental patients as an innovation to reduce T2DM-related multi-morbidity disparities.

### Data collection

An implementation Planning Team of SCF clinical and administrative staff from primary care and dental services proposed the sample populations and recruitment strategies for surveys and individual interviews. The Planning Team recommended adult patients at the SCF dental clinic be sampled for the quantitative inquiry via survey, as they are the potential recipients of the innovation. The CFIR guided development of survey items and responses. The Planning Team also recommended dental and primary care clinical providers, and system leaders be sampled for qualitative inquiry via one-on-one interviews as they are the potential deliverers of the innovation. The CFIR also guided development of semi-structured interview questions and probes for use in individual, in-person interviews. The Planning Team co-developed all data collection instruments prior to data collection. Surveys were collected from April through May 2023, while interviews occurred from February through May 2023.

#### Quantitative data

Survey inclusion criteria were individuals who self-identified as AN/AI, aged 18 years or older, and were eligible to receive care at SCF. The survey sample was recruited in-person by trained SCF research staff placed in SCF Dental Clinic lobbies located on the Alaska Native Health Campus in Anchorage, AK.

The survey consisted of 13-items: five yes/no (e.g., do you have diabetes, do you get regular check-ups at the dental clinic, etc.), six Likert scale, two multiple-choice, and one open-ended response focused on assessing factors that impact patient reach (e.g., CFIR constructs such as patient knowledge and beliefs about T2DM risk and acceptability of receiving T2DM screening in the dental setting). Surveys were self-administered on an iPad with automated data uploaded to a Research Electronic Data Capture (REDCap) database or completed on a paper copy that study staff entered into REDCap [33, 34]. Survey participants were given a $15 gift card in recognition for their time.

#### Qualitative data

Individuals were recruited by email using purposive criterion sampling of SCF dental and primary care providers, staff, and operational leaders recommended by the Planning Team because they are the population of potential innovation deliverers. Inclusion criteria were adults (>18 years old) who interact with patients during the care process or an administrator who oversees clinic operation in the Dental or Primary Care clinics. Individual interviews followed a semi-structured interview guide to understand factors relevant to adoption, implementation, and maintenance of the T2DM screening and referral service in dental clinics.

A trained SCF research team member conducted in-person interviews on the Alaska Native Health Campus in Anchorage, Alaska during clinic hours. Interviews were audio-recorded and transcribed verbatim. Participation was voluntary. Participants were provided SCF promotional items (e.g., t-shirt, knit cap, water bottle) rather than monetary compensation.

### Data analysis

#### Quantitative data

Survey data were exported from REDCap and analyzed using R software v.4.3.1 [35]. The SCF research team conducted univariate statistics and differences between strata were analyzed using Chi square tests for categorical data and *t-*tests for continuous data. Differences with *p* < 0.05 were considered statistically significant.

#### Qualitative data

Analysis of interviews relied upon the TDF guidance for analyzing qualitative data. The updated CFIR guided thematic analysis with attention to inner and outer settings, individual characteristics, innovation processes and innovation characteristics. Interview transcripts were coded using template analysis [36, 37], with initial coding for main themes carried out by JAB and TT. The template was developed further by team member VYH. We defined top-level codes by the major CFIR domains, then subdivided into one or two further coding levels by TDF domain and emergent codes. VYH developed integrative themes within each of the CFIR domains, and identified aspects of the themes as barriers, facilitators or a mix of both barriers and facilitators with illustrative quotations.

#### Convergent analysis

Following the completion of the quantitative and qualitative analyses, the research team conducted convergent analysis. VYH kept an interpersonal, methodological and contextual reflexive journal [38] throughout the qualitative and convergent analysis processes. We compared and contrasted findings from both data types to identify areas of convergence, divergence, or complementarity [39, 40]. We conducted member checking of our convergent analysis findings with the Planning Team so they could be used to develop a clearly defined innovation and innovation support materials for future testing.

### Human Ethics and consent to participate

This study was performed in accordance with the Declaration of Helsinki. This human study was approved by Alaska Area Institutional Review Board (approval: 2017–02-004), and community level approval from the Alaska Native Tribal Health Consortium and Southcentral Foundation. Survey and interview participants provided verbal informed consent prior to participation in data collection.

## Results

### Quantitative summary findings

Two hundred and fifty potential innovation receivers provided survey responses. Most survey participants were female (70%) and had attended college (51%). All were of AN/AI heritage (100%) (Table 2). More than 20% of survey respondents reported not getting regular primary care and 31% of respondents reported not getting regular dental care.

**Table 2:** Characteristics of dental patients surveyed in 2023 regarding screening for type 2 diabetes in a Tribal health organization dental clinic

Sample Characteristics (N)	n (%)
Diabetes Diagnosis	
Yes	22 (9%)
No	190 (78%)
Don’t know	33 (13%)
Participants	250 (100%)
Age, years (n = 246)	
18-29	24 (10%)
30-39	43 (17%)
40-49	57 (23%)
50-59	55 (22%)
60-69	48 (20%)
70+	19 (8%)
Female (n = 242)	170 (70%)
Martial Status (n = 243)	
Single	143 (62%)
Married/ Long-Term Relationship	88 (38%)
Race* (n = 246)	
Alaska Native or American Indian	242 (97%)
White	35 (14%)
Asian	3 (1%)
African American	3 (1%)
Native Hawaiian or Pacific Islander	1 (0.4%)
Education Level (n = 245)	
Less than High School	15 (6%)
High School or GED	87 (36%)
Some College	71 (29%)
Associates or Technical/ Vocational	28 (12%)
Bachelor’s Degree	27 (11%)
Post-Graduate	13 (5%)
Housing Status (n = 244)	
Own or Rent	182 (80%)
Homeless or Temporary Housing	28 (12%)
Other	18 (8%)
Yearly income before taxes (n = 239)	
Less than $15,000	70 (34%)
$15,000-$70,000	104 (50%)
Greater than $70,000	34 (16%)

*Respondents were able to check more than one racial grouping demographic category

Most survey respondents (87%) agreed or strongly agreed that it is important to get screened for T2DM (Table 2). Additionally, the majority (82%) agreed or strongly agreed that the dental clinic is a good place to get T2DM screening. The mean response to the question, “Overall, on a scale of 1 to 7, with 1 being not helpful and 7 being very helpful, how would you rate the helpfulness in being screened for diabetes in the dental setting.”, was 6 (SD = 1.3). When considering diabetes screening in the dental clinic, benefits included convenience (55%), saving time (54%), and talking to trained office staff during a dental visit would provide them with information to stay healthy (43%). Only 10% of respondents did not think getting screened for T2DM at the dental clinic would be helpful to them.

Two thirds of participants had no concerns about screening for diabetes in the dental setting. When asked, “Overall, on a scale of 1 to 7, with 1 being not concerned at all and 7 being very concerned, how would you rate your level of concern in being screened for diabetes in the dental setting.”, the mean response was 3.2 (SD = 2.2). Of those who reported concerns, 12% were concerned the dental staff were not trained to provide them with T2DM prevention information, 11% were concerned that dental staff are not qualified to conduct this type of screening, 8% were concerned their T2DM screening results may not be shared with their primary care provider, 8% were concerned someone would see their screening result who should not, 8% were concerned with inaccurate test results and 3% were concerned with unnecessary pain or risk of infection.

### Qualitative summary findings

Ten staff (seven dental; three primary care) participated in the individual interviews. Interview duration ranged from 22 to 52 minutes. Most of the interview participants were female (70%), not Hispanic or Latino (90%), and White (70%). Nearly a third of interviewees (30%) identified as being of AN/AI heritage. Interview participants reported having five or more years employment and having leadership roles as members of Tribal health organization’s clinical, operational, and administrative leadership committees (70%).

Dental staff described interest in the innovative concept of screening for T2DM in the dental setting, whereas primary care staff expressed reluctance. Primary care staff questioned whether T2DM screening in dental would fill an existing need. Primary care staff expressed that when patients come into primary care, they will get T2DM screening. They did not think a subset of the AN/AI population visited the dental clinic without visiting the primary care clinic. However, dental staff acknowledged that there are patients who visit the dental clinic and not the primary care clinic, as a dental provider/leader said:

*I think it could fill an existing health need. I think that there are customer-owners [patients] who choose to access one portion of our healthcare system more frequently than others … this could be an opportunity to expand our prevention efforts, or at least educational efforts. *(P4)

Primary care staff expressed that all AN/AI people eligible for health care are routinely screened for T2DM and would like to see data to support idea that there are gaps in screening.

Interview participants frequently discussed possible screening process issues. Dental staff suggested that screening questions to identify risk factors should take place when dental patients (innovation recipients) checked into dental clinic and questioned which dental provider would be asking those questions and providing additional information based on the T2DM screening response. Suggestions on a T2DM risk factor screening process included adding screening questions to a pre-existing intake form or having dental staff review a patient’s electronic health record. Dental staff also asked who would review risk factor information and determine if the patient was eligible for point-of-care HbA1c testing. When positing that once an individual was determined to have at least two risk factors and had not been screened in the last 12 months, some dental staff questioned who was going to conduct the finger prick. It was noted that primarily, the dental assistant and the dental hygienist gather medical history information at each appointment, so these positions were suggested as potentially being responsible for performing risk factor screening. Some dental staff also noted that the dental assistant or dental hygienist could perform the finger prick, and it could be performed before or after they measure the patient’s blood pressure, which is routinely screened before dental procedures.

Additionally, questions arose from both dental and primary care staff about who would be reading the results to the patient and where and when this would take place. Mainly, participant concerns were whether dental staff have the background to answer questions about diabetes and the amount of dental staff time this would take. All dental staff noted time constraints as a concern for adding diabetes screening to dental visits. Result documentation in the electronic health record was a concern that arose among both primary care and dental staff. Most dental staff also noted that there was no current protocol in place to refer dental patients to their primary care provider and most primary care staff noted that primary care teams do not have a protocol in place to receive referrals from other providers within the healthcare system. Dental staff described a need for a process to communicate a T2DM screening result to the patients’ primary care team.

### Consolidated framework for implementation research and theoretical domains framework themes

Fourteen TDF domains were coded from the interviews. The most frequently coded TDF domains were memory, attention and decision processes, and social influences (30 quotations total); whereas the domains of beliefs about capabilities, optimism, beliefs about consequences, and reinforcement domains had no coded quotations. (Supplemental Table 1).

**Fig. 1:**
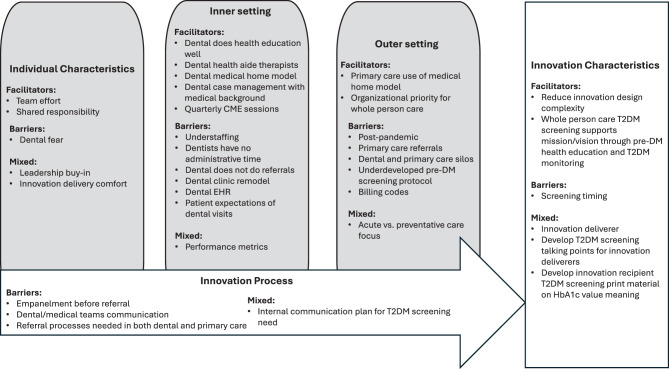
CFIR constructs and their influence on pre-implementation

Drivers of possible success or failure of innovation implementation described in interviews were associated with the five major CFIR domains of innovation characteristics, individual characteristics, inner setting, outer setting and implementation process (Fig. 1. CFIR Constructs and their influence on pre-implementation). We describe the barriers and facilitators by the major CFIR domain below and provide the CFIR domains, associated TDF themes and illustrative quotations in Table 3.

**Table 3:** Barriers and facilitators by consolidated framework for implementation research (CFIR) domains with theoretical domains framework (TDF) and illustrative quotes

CFIR domains	TDF related themes	Illustrative quote
Individual characteristics	Social/professional role and identity, beliefs about capabilities, optimism, and emotion	“I think there, there’s going to be resistance, within the department because there’s just a feeling that we don’t have time to do this and this is just an ask and, there might be ideas like, well, could this just be done kind of at a booth in the waiting area instead of, you know, chair side with kind of very individualized conversations with the customer [patient]? I think that’s, that’s clearly going to be some I – some people have that ideas that maybe that’s a better approach instead of really taking up all the dentists’ or the hygienists’ or the DAs’ [dental assistant’s] time. Not all of it, but some portion of it.” (P2)
Inner setting	Intentions and goals	“Time is the biggest challenge, and then training, getting providers familiar and comfortable, having that conversation, and doing the finger prick and all of that with the equipment. And then just with the challenge of time, preventing it from being one of the things that falls to the wayside, and really prioritizing it, and make sure it gets included at each visit” (P5)
Outer setting	Environmental context and resources	“I’m not aware of any referrals from dental to primary care. … I’m not aware of any referrals that primary care gets. … I think that would really depend operationally on staffing in both locations [dental and primary care], what access looks like in both locations, what really the priorities are for both divisions in terms of meeting customer-owner [patient] needs, where for me as a director in primary care, a concern I have is anything that diverts our staff’s attention away from just being able to provide really timely, comprehensive care to our customer-owners [patients]. And so, managing referral queues, that’s something that could potentially do that. And then it also potentially is one of those things that opens the door to other referrals, where really again it’s that we should maintain open access for customers [patients]. They should be able to get their needs met comprehensively, holistically, and to me, that doesn’t require a referral.” (P12)
Innovation process	Knowledge, skills, memory, attention and decision processes, and social influences	“There would need to be buy-in, at our leadership level, medical directors, core business groups, and then I would have to go to the disciplines, and then there comes a decision of, who owns this, and at what cost? Because if my job goes from being really big, ‘cause I’m not just – I’m doing cancer screenings. I’m doing triaging of sick babies and trying to get immunizations and outreach and pull people in and manage diabetes care as well as I can, and make sure that we’re utilizing all of our resources for customer owners and for the 1,100–1,200 customer-owners [patients] on my panel, I’m looking at the metrics. How are we doing? And now we’re throwing in another part of your job is managing a referral queue, something’s gonna fall. And what can fall?.” (P12)
Innovation/Intervention characteristics	Beliefs about consequences	“In many ways, our customer owners become frustrated with their other health issues and how they relate to their dental care. And I think it’s hard for them to make that connection, but if we constantly remind our customer owners about how their diet and how diabetes, specifically, can affect their overall health, and if we’re persistent about it, I think can end up being very fruitful initiative.” (P7)

### Individual characteristics

Within the individual characteristics domain, facilitators focused on identifying potential innovation deliverers by dental team role. Dentists were identified as mid-level leaders; however, no distinct role was clearly identified as the implementation facilitator., Rather, multiple members of the dental care team were identified as deliverers, making the innovation a team effort. Both dental and primary care providers also noted the need for innovation recipient choice and deliverer discretion in screening, that a patient should be able to opt out of the screening, particularly if they were experiencing dental fear. Additionally, a provider should be able to prioritize other aspects of patient care, mostly if the patient was presenting with acute pain or with a dental emergency.

### Inner setting

Major barriers to implementation of a T2DM screening process were infrequent collaboration between dental and primary care providers, and no routine procedure for primary care to receive referrals from other sectors of the health system. The major inner setting facilitator was the local attitudes of staff in both dental and primary care, as they focused on leveraging the medical home model in both settings to support their shared responsibility in helping recipients and upholding beliefs of moving towards whole-person care. Within the dental clinic, the ongoing improvement activities in the organization of tasks and responsibilities of members of the dental care team following the transition to a dental medical home model and during recovery from the COVID-19 pandemic were core issues within the work infrastructure. Additionally, limitations of the dental electronic health record were identified as barriers to monitoring innovation outcomes and supporting functional performance within the dental clinic. The major concerns noted by all dental staff interviewees were the interconnected barriers of time and relative priority within a dental visit, and the staffing issues of training and understaffing of clinical positions due to workforce shortages.

Factors that could facilitate innovation implementation were focused on the culture of the dental clinic, with a shared value, belief and norm of centering the dental environment and patient encounters on caring for, supporting, and addressing the needs and wellbeing of AN/AI peoples through whole-person care. One dental provider stated:

*I think SCF does a good job at acknowledging that health is more than just what a customer [patient] presents for that day. It is whole person health, not just physical, but as it states in our vision and our mission about physical, mental, emotional, and spiritual well-being. *(P4)

### Outer setting

The partnerships and connections within the SCF healthcare system were the focus of discussion along with policies and professional licensure regulations and professional group silos (e.g., the historic differentiation between dental and medical care).

### Innovation process

Intentional coordination and collaboration on interdependent tasks in the screening process were major areas of discussion related to innovation processes. For example, a primary care interviewee described communication improvements needed within various levels of the health system for implementation buy-in and innovation process approval, as well as managing competing population health priorities within the medical home panel. Also, within the innovation process domain were discussions on planning such as defining goals and measures for innovation success in advance.

### Innovation/Intervention characteristics

The design, roll-out, and trialability of the innovation were major areas of discussion on innovation characteristics. Respondents described an interest in an innovation that had a minimal number of steps, limited scope, clear deliverer messaging with consistency in training of message deliverers, and reinforcement of messages through written health education handouts for innovation recipients.

### Convergent results

The survey and interview summary findings were presented to the implementation Planning Team for their consideration, with conversation facilitated by the research team focused on issues within the innovation process and innovation characteristics CFIR domains. Although dental and primary care interviewees mentioned having concerns about patient interest in receiving T2DM screening in the dental setting or by dental staff due to T2DM screening not usually occurring in a dental visit, the majority of patient survey respondents thought the dental clinic as a good place to get screened for diabetes and had low levels of concern about being screened for T2DM in dental settings. Moreover, while primary care staff did not see the need for T2DM screening in dental settings, nearly half of survey respondents thought the T2DM screening information would be helpful for their doctor, with half of the respondents thinking the T2DM screening in dental would be a good way to find if they were at risk for T2DM and know if they currently have T2DM. Both sample groups, desired handouts or other print materials for health education purposes explaining the need for T2DM screening, T2DM risk factors, and T2DM screening results.

## Discussion

The potential of dental health visits as a vehicle for expanding the reach of preventive services for individuals at risk for T2DM is well documented in the literature [41, 42]. However, implementation of diabetes screening as a component of routine oral health practice, along with increased care coordination between dental and primary care, has not been widely adopted within real world practice [43]. This convergent parallel mixed-methods study identified community support for T2DM screening in dental clinics, with whole person care and time-saving as top benefits. However, both potential innovation recipients and deliverers expressed concerns about results communication and staff training, and dental staff worried about the time to conduct T2DM screening within the scheduled appointment time block. Also, while interviewed dental staff acknowledged the potential for T2DM screening in dental to meet the needs of patients who do not regularly visit primary care, primary care interviewees did not universally endorse a need for additional screening for T2DM outside of the primary care practice.

The healthcare quality improvement literature promotes the importance of better integration and care coordination between dentistry and primary medicine to address common risk factors for disease, given known linkages between oral and systemic health [44]. A recent systematic review of the literature indicated several models for integrating dental and primary care including co-location of oral health professionals in primary care clinics, care coordination and referrals, and full integration of dental services into the medical home [45, 46]. Some known benefits of integrating medical and dental services include addressing access issues in rural communities as well as gaps in care across the lifespan, and yet professional resistance and system-level issues (e.g., barriers to data sharing and lack of referral systems) persist as obstacles to aligning dental and primary care services [47].

In our study, the lack of a referral process from dental to primary care clinics’ provider teams was confirmed as a major barrier. Although both dental and primary care systems utilize home models and practice whole-person care with integrated care team members available to support case management, there is no process in place for referrals from a dental home team to a primary care medical home team. Historic professional barriers were also acknowledged between the dental and medical professions, with regard to beliefs about roles, academic preparation, clinical training, and professional licensing and state/national policy pertaining to scope of practice and financing of health care services; concerns that are consistent with other studies [48]. On the other hand, there was strong dental staff interest in being more integrated with primary care and all interviewed staff embraced the focus on whole person health in a health care system based on AN/AI values, and AN/AI identified health priorities. Thus, the opportunity to achieve common goals of preventing or mitigating risk factors associated with T2DM preventing complications aligns with system-wide values. To achieve true whole person health will require a shift of professional attitudes and beliefs about the value of integrated dental health services, such as T2DM or pre-diabetes screening, and acceptance of a structured dental referral service to assure appropriate follow-up assessment and care.

Studies identifying barriers to coordinating dental and primary care services are limited, with few using implementation science frameworks to guide pre-implementation data collection from key stakeholders to understand the multi-level contextual factors relevant to implementation of practice change [49]. This is one of only a few studies that have included input from patients, dental professionals, and primary care providers to identify pre-implementation barriers and facilitators important for co-development of integrated practice change [50]. A key strength was our use of implementation frameworks, as recommended by Moullin et al. [51], during all stages of our study, including using CFIR constructs to develop qualitative and quantitative data collection instruments relevant to implementation and sustainment of the innovation [52]. Construct selection was validated by the multi-disciplinary Planning Team familiar with both the inner and outer settings of the innovation location, current T2DM screening practices, and systems. Additionally, the research team presented the preliminary findings to the Planning Team for their interpretation of themes, innovation process needs, and innovation characteristics. Finally, the investigator who performed the coding and convergent analysis kept a reflexive journal to improve the rigor and trustworthiness of the results through transparency about the investigator’s coding and convergent analysis process.

Our study also has several limitations. The study relied upon voluntary participation, which may have introduced selection bias, resulting in themes not being applicable to the general AN/AI population served at SCF dental or the staff who would be impacted by the innovation. Our survey used a convenience sample, and we used purposive sampling in our interview recruitment. Due to the recruitment processes and the healthcare setting, we may have findings with a non-response bias as well as an acquiescence bias. By using a survey with potential intervention recipients and interviews with potential innovation deliverers, we were not able to directly compare respondent views. A single investigator conducted most of the coding, which might have affected the rigor of our findings as other investigators may have coded responses differently and identified different themes. However, a benefit of using a single coder is efficiency and consistency of coding. Despite the qualitative and quantitative data instruments being developed using CFIR, all CFIR domains did not directly overlap across the instruments. This being the case, some areas of inquiry may not have been addressed in both samples. Finally, CFIR provides a static representation of context, and as healthcare environments are dynamic, our findings may miss evolving implementation conditions.

## Conclusion

Creating a dental-primary care referral service may open doors for improved communication between dental and primary care providers, increase the number of contact points for screening, and distribute the burden of addressing T2DM among multiple provider types. It affords an additional opportunity to identify persons at risk prior to disease onset, maximizing the ability to arrest T2DM progression *before* complications become irreversible.

Overall, the results of this study emphasize the importance of pre-implementation data collection from innovation recipients and innovation deliverers to inform the design of a relevant and feasible innovation. Our findings will be used by the project’s Planning Team to develop an innovation and devise strategies to implement it. In addition, these results provide a framework for proactively identifying and addressing barriers and facilitators prior to innovation implementation to maximize the fit and feasibility of implementing evidence-based innovations in real-world settings.

## Electronic supplementary material

Below is the link to the electronic supplementary material.

Supplementary material 1

## Data Availability

The datasets analyzed during the current study are available from the corresponding author upon approval from the Alaska Native Tribal Health Consortium and Southcentral Foundation.

## Abbreviations

T2DM: Type 2 diabetes mellitus
CFIR: Consolidated framework for implementation research
TDF: Theoretical domains framework
AN/AI: Alaska Native and American Indian
PY: Person-years
SCF: Southcentral Foundation
REDCap: Research Electronic Data Capture

## References

[1] Duan D, Kengne AP, Echouffo-Tcheugui JB. Screening for diabetes and prediabetes. Endocrinol Metab Clinics N Am. 2021;50(3):369–85. 10.1016/j.ecl.2021.05.002. 10.1016/j.ecl.2021.05.002 PMC8375583 34399951

[2] Heron M. Deaths: leading causes for 2017. Natl Vital Stat Rep. 2019;68(6):1–77. 32501203

[3] Lanier AP, Redwood DG, Kelly JJ. The Alaska Education and research Towards health (EARTH) study: cancer risk factors. J Canc Educ. 2012;27(S1):S80–85. 10.1007/s13187-012-0333-4. 10.1007/s13187-012-0333-4 PMC9888362 22298198

[4] Slattery ML, Ferucci ED, Murtaugh MA, Edwards S, Ma K-N, Etzel RA, et al. Associations among body mass index, waist circumference, and health indicators in American Indian and Alaska Native adults. Am J Health Promot. 2010;24(4):246–54. 10.4278/ajhp.080528-QUAN-72. 20232606 10.4278/ajhp.080528-QUAN-72 PMC2925498

[5] Kim SY, England LJ, Shapiro-Mendoza CK, Wilson HG, Klejka J, Tucker M, et al. Community and Federal collaboration to assess pregnancy outcomes in Alaska Native women, 1997–2005. Matern Child Health J. 2014;18(3):634–39. 10.1007/s10995-013-1287-9. 23775248 10.1007/s10995-013-1287-9 PMC4392760

[6] Redwood DG, Lanier AP, Johnston JM, Asay ED, Slattery ML. Chronic disease risk factors among Alaska Native and American Indian people, Alaska, 2004–2006. Prev Chronic Dis. 2010;7(4):A85. PMC2901583 20550843

[7] Beans JA, Hiratsuka VY, Shane AL, Day GE, Redwood DG, Flanagan CA, et al. Follow-up study methods for a longitudinal cohort of Alaska Native and American Indian people living within urban South Central Alaska: the EARTH study. J Community Health. 2019;44(5):903–11. 10.1007/s10900-019-00630-z. 30798425 10.1007/s10900-019-00630-z PMC6707895

[8] Koller KR, Day GE, Hiratsuka VY, Beans JA, Nash SH, Redwood DG, et al. Increase in diabetes among urban Alaska Native people in the Alaska EARTH follow-up study: a call for prediabetes screening, diagnosis, and referral for intervention. Diabetes Res Clin Pr. 2020;167:108357. 10.1016/j.diabres.2020.108357. 10.1016/j.diabres.2020.108357 PMC7530054 32745696

[9] Glasgow RE, Emmons KM. How can we increase translation of research into practice? Types of evidence needed. Annu. Rev. Public Health. 2007;28(1):413–33. 10.1146/annurev.publhealth.28.021406.144145. 17150029 10.1146/annurev.publhealth.28.021406.144145

[10] Hiratsuka VY, Moore L, Dillard DA, Avey JP, Dirks LG, Beach B, et al. Development of a screening and brief intervention process for symptoms of psychological trauma among primary care patients of two American Indian and Alaska native health systems. J Behav Health Serv Res. 2017;44(2):224–41. 10.1007/s11414-016-9519-6. 27328846 10.1007/s11414-016-9519-6 PMC5177536

[11] Prestwich A, Sniehotta FF, Whittington C, Dombrowski SU, Rogers L, Michie S. Does theory influence the effectiveness of health behavior interventions? meta-analysis. Health Phychol: Off J The Div Health Phychol, Am Psychological Assoc. 2014;33(5):465–74. 10.1037/a0032853. 10.1037/a0032853 23730717

[12] Michie S. Designing and implementing behaviour change interventions to improve population health. J Health Serv Res Policy. 2008;13 Suppl, 3: 3_suppl):64–69. 10.1258/jhsrp.2008.008014. 18806194 10.1258/jhsrp.2008.008014

[13] Cane J, O’Connor D, Michie S. Validation of the theoretical domains framework for use in behaviour change and implementation research. Implementation Sci : IS. 2012;7(1):37. 10.1186/1748-5908-7-37. 22530986 10.1186/1748-5908-7-37 PMC3483008

[14] O’Donovan B, Kirke C, Pate M, McHugh S, Bennett K, Cahir C. Mapping the theoretical domain framework to the consolidated framework for implementation research: do multiple frameworks add value? Implement Sci Commun. 2023;4(1):100. 10.1186/s43058-023-00466-8. 37620981 10.1186/s43058-023-00466-8 PMC10464139

[15] Damschroder LJ, Aron DC, Keith RE, Kirsh SR, Alexander JA, Lowery JC. Fostering implementation of health services research findings into practice: a consolidated framework for advancing implementation science. Implementation Sci : IS. 2009;4(1):50. 10.1186/1748-5908-4-50. 19664226 10.1186/1748-5908-4-50 PMC2736161

[16] Schoonenboom J, Johnson RB. Wie man ein mixed methods-forschungs-design konstruiert. Köln Z Soziol. 2017;69(Suppl S2):107–31. 10.1007/s11577-017-0454-1. 10.1007/s11577-017-0454-1 PMC5602001 28989188

[17] Koller K, Beans J, King D, Hiratsuka VY, Day GM, Strauss SM, et al. Pilot test of diabetes screening and referral to primary care within an Alaska Native dental clinic: protocol for a Feasibility study. 2026. JMIR Res Protocol. 2026;15:e75619. 10.2196/75619. 10.2196/75619 PMC12978979 41813430

[18] Minkler MWN. Community based participatory research for health. San Francisco, CA: Jossey-Bass; 2003.

[19] Mariella PBE, Carter M, Verri V. Tribally-driven participatory research: state of the practice and potential strategies for the future. J Health Dispar Res Pract. 2009;3(2):41–58.

[20] Cox GR, FireMoon P, Anastario MP, Ricker A, Escarcega-Growing Thunder R, Baldwin JA, et al. Indigenous standpoint theory as a theoretical framework for decolonizing social science health research with American Indian communities. AlterNative: Int J Indigenous Peoples. 2021;17(4):460–68. 10.1177/11771801211042019. 10.1177/11771801211042019 PMC11046738 38680293

[21] Ray L, Hiratsuka VY, Cheung K, Dillard DA, Tierney M, Manson SM. Making a community health needs assessment participatory: a case study from an Alaska Native health care organization. AIANMHR. 2025;32(2):1–26. 10.5820/aian.3202.2025.1. 10.5820/aian.3202.2025.1 40106824

[22] Hiratsuka VY, Beans JA, Robinson RF, Shaw JL, Sylvester I, Dillard DA. Self-determination in health research: an Alaska Native example of Tribal ownership and research regulation. IJERPH. 2017;14(11):1324. 10.3390/ijerph14111324. 29088111 10.3390/ijerph14111324 PMC5707963

[23] Strauss SM, Russell S, Wheeler A, Norman R, Borrell LN, Rindskopf D. The dental office visit as a potential opportunity for diabetes screening: an analysis using NHANES 2003–2004 data. J Public Health Dent. 2010;70(2):no-no. 10.1111/j.1752-7325.2009.00157.x. 10.1111/j.1752-7325.2009.00157.x 20002875

[24] Strauss SM, Alfano MC, Shelley D, Fulmer T. Identifying unaddressed systemic health conditions at dental visits: patients who visited dental practices but not general health care providers in 2008. Am J Public Health. 2012;102(2):253–55. 10.2105/AJPH.2011.300420. 22390440 10.2105/AJPH.2011.300420 PMC3483998

[25] Strauss SM, Rosedale M, Pesce MA, Juterbock C, Kaur N, DePaola J, et al. Point-of-care HbA1c testing with the A1cNow test Kit in general practice dental clinics: a pilot study involving Its accuracy and practical issues in Its use. Point Care: J Near-Patient Test Technol. 2014;13(4):142–47. 10.1097/POC.0000000000000039. 10.1097/POC.0000000000000039 PMC4290166 25593546

[26] Wright D, Muirhead V, Weston-Price S, Fortune F. Type 2 diabetes risk screening in dental practice settings: a pilot study. Br Dent J. 2014;216(7):E15. 10.1038/sj.bdj.2014.250. 10.1038/sj.bdj.2014.250 24722119

[27] Strauss SM, Stefanou LB. Interdental cleaning among persons with diabetes: relationships with individual characteristics. Int J Dent Hyg. 2014;12(2):127–32. 10.1111/idh.12037. 23790179 10.1111/idh.12037

[28] Strauss SM, Singh G, Tuthill J, et al. Diabetes-related knowledge and sources of information among periodontal patients: is there a role for dental hygienists? J Educ Chang Dent Hyg: JDH / Am Dent Hygienists’ Assoc. 2013;87(2):82–89. PMC4143392 23986141

[29] Damschroder LJ, Reardon CM, Widerquist MAO, Lowery J. The updated consolidated framework for implementation research based on user feedback. Implementation Sci : IS. 2022;17(1):75. 10.1186/s13012-022-01245-0. 36309746 10.1186/s13012-022-01245-0 PMC9617234

[30] French SD, Green SE, O’Connor DA, McKenzie JE, Francis JJ, Michie S, et al. Developing theory-informed behaviour change interventions to implement evidence into practice: a systematic approach using the theoretical domains framework. Implementation Sci : IS. 2012;7(1):38. 10.1186/1748-5908-7-38. 22531013 10.1186/1748-5908-7-38 PMC3443064

[31] Driscoll DL, Hiratsuka V, Johnston JM, Norman S, Reilly KM, Shaw J, et al. Process and outcomes of patient-centered medical care with Alaska Native people at Southcentral Foundation. Ann Fam Med. 2013;11 (Suppl 1): Suppl_1:S41–49. 10.1370/afm.1474. 10.1370/afm.1474 PMC3707246 23690385

[32] Girish Babu KL, Doddamani GM. Dental home: Patient centered dentistry. J Int Soc Prevent Communit Dent. 2012;2(1):8–12. 10.4103/2231-0762.103448. 10.4103/2231-0762.103448 PMC3894088 24478960

[33] Harris PA, Taylor R, Thielke R, Payne J, Gonzalez N, Conde JG. Research electronic data capture (REDCap)—A metadata-driven methodology and workflow process for providing translational research informatics support. J Retailing Biomed Inf. 2009;42(2):377–81. 10.1016/j.jbi.2008.08.010. 10.1016/j.jbi.2008.08.010 PMC2700030 18929686

[34] Harris PA, Taylor R, Minor BL, Elliott V, Fernandez M, O’Neal L, et al. The REDCap consortium: building an international community of software platform partners. J Retailing Biomed Inf. 2019;95:103208. 10.1016/j.jbi.2019.103208. 10.1016/j.jbi.2019.103208 PMC7254481 31078660

[35] *R: a language and environment for Statistical Computing*. [computer program]. Version version 4.3.1. Vienna, Austria: R Foundation for Statistical Computing; 2024.

[36] Crabtree BF, Miller WL. Using codes and code manuals: a template organizing style of interpretation. In: Crabtree BF, Miller WL, editors. Doing qualitative research. 2nd. Newbury Park, CA: SAGE, Publications Inc; 1999. p. 163–78.

[37] King N Doing Template Analysis. in: Symon, G, Cassell, C, eds. Qualitative organizational research: core methods and current challenges. SAGE Publications, Inc; 2012. p. 426–50.

[38] Olmos-Vega FM, Stalmeijer RE, Varpio L, Kahlke R. A practical guide to reflexivity in qualitative research: AMEE guide No. 149. Med Teach. 2023;45(3):241–51. 10.1080/0142159X.2022.2057287. 10.1080/0142159X.2022.2057287 35389310

[39] Zhang W, Creswell J. The use of “mixing” procedure of mixed methods in health services research. Med Care. 2013;51(8):e51–57. 10.1097/MLR.0b013e31824642fd. 10.1097/MLR.0b013e31824642fd 23860333

[40] Katz-Buonincontro J. How to mix methods: a guide to sequential, convergent, and experimental research designs. In: Katz-Buonincontro J, editor. *Convergent mixed methods designs*.Source American Psychological Association;. 2024. p. 73–82.

[41] Priede A, Lau P, Mariño R, Darby I. Enhancing diabetes screening among oral healthcare professionals: a COM-B model and a theoretical domains framework approach. 2025. Diabetology. 2025;6(10):113. 10.3390/diabetology6100113.

[42] Zelig R, Samavat H, Duda P, Singer S, Feldman C, LaSalle P, et al. Screening for diabetes risk using the diabetes risk test and point-of-care hemoglobin A1C values in adults seen in a dental clinic. Quintessence Int (Berl) (Berlin, Ger : 1985). 2023;54(6):500–09. 10.3290/j.qi.b3957685. 10.3290/j.qi.b3957685 36917464

[43] Doughty J, Mg S, Paisi M, Witton R, Jd A. Opportunistic health screening for cardiovascular and diabetes risk factors in primary care dental practices: experiences from a service evaluation and a call to action. Br Dent J. 2023;235(9):727–33. 10.1038/s41415-023-6449-6. 37945870 10.1038/s41415-023-6449-6 PMC10635822

[44] Io M, Council NR. Improving access to oral health care for vulnerable and underserved populations. Washington, DC: The National Academies Press; 2011.

[45] Prasad M, Manjunath C, Murthy AK, Sampath A, Jaiswal S, Mohapatra A. Integration of oral health into primary health care: a systematic review. J Fam Med Prim Care. 2019;8(6):1838–45. 10.4103/jfmpc.jfmpc_286_19. 10.4103/jfmpc.jfmpc_286_19 PMC6618181 31334142

[46] Patil PD, Jadhav PS, K S, U PG, D K, Maruthavanan R. Linking systemic diseases with oral health: awareness among general physician. Bioinformation. 2025;21(4):841–44. 10.6026/973206300210841. 40636167 10.6026/973206300210841 PMC12236542

[47] Vermillion M, Kanan C, McLeod C, Chokas C, Clark R, Clark A, et al. Scaling medical-dental integration nationally: outcomes from the MORE care initiative. Front Public Health. 2026;14:1753528. 10.3389/fpubh.2026.1753528. 41788526 10.3389/fpubh.2026.1753528 PMC12956783

[48] Mariño R, Priede A, King M, Adams GG, Lopez D. Attitudes and opinions of oral healthcare professionals on screening for type-2 diabetes. BMC Health Serv Res. 2021;21(1):743. 10.1186/s12913-021-06756-y. 34315460 10.1186/s12913-021-06756-y PMC8314562

[49] Spallek H, Song M, Polk DE, Bekhuis T, Frantsve-Hawley J, Aravamudhan K. Barriers to implementing evidence-based clinical guidelines: a survey of early adopters. J Evidence Based Dent Pract. 2010;10(4):195–206. 10.1016/j.jebdp.2010.05.013. 10.1016/j.jebdp.2010.05.013 PMC3011934 21093800

[50] Brocklehurst PR, Williams L, Burton C, Goodwin T, Rycroft-Malone J. Implementation and trial evidence: a plea for fore-thought. Br Dent J. 2017;222(5):331–35. 10.1038/sj.bdj.2017.213. 28281585 10.1038/sj.bdj.2017.213

[51] Moullin JC, Dickson KS, Stadnick NA, Albers B, Nilsen P, Broder-Fingert S, et al. Ten Recommendations for using implementation frameworks in research and practice. Implement Sci Commun. 2020;1(1):42. 10.1186/s43058-020-00023-7. 32885199 10.1186/s43058-020-00023-7 PMC7427911

[52] Damschroder LJ. Clarity out of chaos: use of theory in implementation research. Psychiatry Res. 2020;283:112461. 10.1016/j.psychres.2019.06.036. 31257020 10.1016/j.psychres.2019.06.036

