# Supplementary material for: Barriers and facilitators at pre-implementation of a type 2 diabetes screening program in dental care using the consolidated framework for implementation research and the theoretical domains framework
Source: BMC Health Serv Res. 2026 Jun 6;26:1112. doi: 10.1186/s12913-026-14902-7 (PMC13471304; doi:10.1186/s12913-026-14902-7)
Supplement: Supplementary file 1 [file 12913_2026_14902_MOESM1_ESM.docx]

**Supplemental Table 1.**

*TDF Domains and Quotations*

| TDF domain / CFIR antecedent assessment code | *n* |
| --- | --- |
| Memory, attention and decision processes; Social influences | 30 |
| Environmental context and resources | 28 |
| Emotion | 25 |
| Intentions; goals | 22 |
| Behavioral regulation | 16 |
| Social/professional role and identity | 15 |
| Knowledge; skills | 12 |
| Beliefs about capabilities; optimism | 0 |
| Beliefs about consequences | 0 |
| Reinforcement | 0 |
| Total | 148 |
